# Preterm infant retinal OCT markers of perinatal health and retinopathy of prematurity

**DOI:** 10.3389/fped.2023.1238193

**Published:** 2023-09-21

**Authors:** Shwetha Mangalesh, Cynthia A. Toth

**Affiliations:** Department of Ophthalmology, Duke University School of Medicine, Durham, NC, United States

**Keywords:** preterm infant, optical coherence tomography, perinatal systemic health, OCT, preterm infant retina, retinal nerve fiber layer, choroid, retinopathy of prematurity

## Abstract

The increasing survival of preterm infants has led to the importance of improving long-term outcomes associated with preterm birth. Antenatal and perinatal insults not only impact mortality, but also long-term disability. While in the intensive care nursery, preterm infants are also exposed to various stressors that lead to long-term cognitive deficits. It is therefore critical to identify early, low-stress, non-invasive biomarkers for preterm infant health. Optical coherence tomography (OCT) is a powerful imaging modality that has recently been adapted to the infant population and provides noninvasive, high-resolution, cross-sectional imaging of the infant eye at the bedside with low stress relative to conventional examination. In this review we delve into discussing the associations between preterm systemic health factors and OCT-based retinal findings and their potential contribution to the development of non-invasive biomarkers for infant health and for retinopathy of prematurity (ROP).

## Introduction

Optical coherence tomography (OCT) imaging has been the standard of care for the diagnosis and management of retinal diseases in adults and children. It provides a high-resolution, cross-sectional view of the retina useful in clinical decision-making. OCT imaging with the tabletop systems was historically limited to adults and school-aged children who could cooperate with the upright chin-rest apparatus. Imaging infants however, was challenging with the tabletop systems owing to the non-compliant design of the system and the inability of the infants to fixate as well as adults (1, 2). Handheld OCT systems have been developed to address this challenge (3). The application of handheld OCT in infants at the bedside has revolutionized our understanding of infant retina by enabling visualization of both the retinal structure and vasculature. We first described the use of handheld OCT to image infants with injury or disease, e.g., shaken baby syndrome (3) and retinopathy of prematurity (ROP) during examination under anesthesia and then at the bedside in the intensive care nursery (ICN) (4, 5). Our application of investigational handheld OCT in infant research in 2008 yielded images and new insights into the microanatomy of the developing retina and choroid (6–8) and especially of the infant fovea and led to a commercial system that became available in 2012 (Envisu 2300, Bioptigen/Leica, Morrisville, NC). This information was not available from conventional clinical examination and included the discovery of unexpected findings in preterm infant eyes including macular edema (9–11), and evidence of diseased vasculature (12), retinal schisis and extent of retinal detachment from ROP (4) and its effects on the developing retina and choroid (13–16). This led to studies by multiple groups into the rapidly changing microanatomy of the preterm infant and neonatal retina after birth and through early years of life as was summarized in recent reviews (17, 18).

We have shown that the wealth of microanatomic information in OCT images of the rapidly developing preterm infant retina is informative of the progression of ROP, retinal and systemic disease and of infant functional and neurodevelopmental outcomes (19–22). Given the implication of preterm birth on long-term neurodevelopmental outcomes, it may be useful to find early biomarkers to predict these outcomes. Preterm infants are also exposed to a myriad of early-life stressors while in the ICN, such as excessive light, eye examination or retinal photographs, and blood draws, and exposure to stress in this period is associated with poorer neurodevelopment (23–25). Hence, a non-stressful, non-invasive clinical tool that could be utilized to assess retinal neurovasculature for ROP and which may also reflect systemic and neurological health and monitor development and treatment responses was developed to address this critical need.

**Figure 1:**
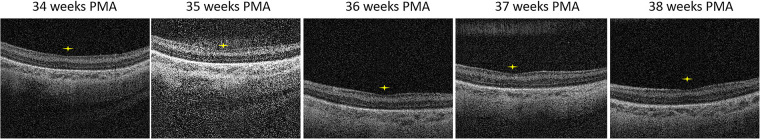
Representative handheld optical coherence tomography (OCT) serial images at the fovea (yellow star) acquired weekly from 34 weeks postmenstrual age (PMA) to 38 weeks PMA from the left eye of the same infant.

While multiple studies have explored the association between infant retina on OCT and ROP (10, 15, 26, 27), we have investigated the relationship between antenatal and perinatal systemic health on the infant retina in sick and healthy term and preterm infants and young children, from 2008 to present, under multiple IRB-approved protocols, and with parent or guardian consent. This has included novel findings in infants with hypoxic ischemic encephalopathy, neonatal hemochromatosis, and infants and young children with retinoblastoma and inherited diseases (9, 22, 28, 29). Our Study of Eye Imaging in Preterm Infants (BabySTEPS; ClinicalTrials.gov: NCT02887157) is a prospective, longitudinal study initiated in 2016 to evaluate normal and abnormal microanatomic features of the developing retina using bedside handheld OCT in very preterm infants at risk for ROP (15). We designed BabySTEPS to address the limitations of previous OCT imaging studies in preterm infants that have not sought to generate serial images within specified time windows (Figure 1) or to measure the sub-layer thicknesses of the developing infant retina (15). Our goal for this review, is to discuss and highlight the method of OCT imaging in the nursery, retinal findings associated with systemic and retinal disease and with ROP, and the potential for these to lead to the development of non-invasive biomarkers for infant systemic and ocular health including ROP.

### Handheld OCT imaging in the ICN

**Figure 2:**
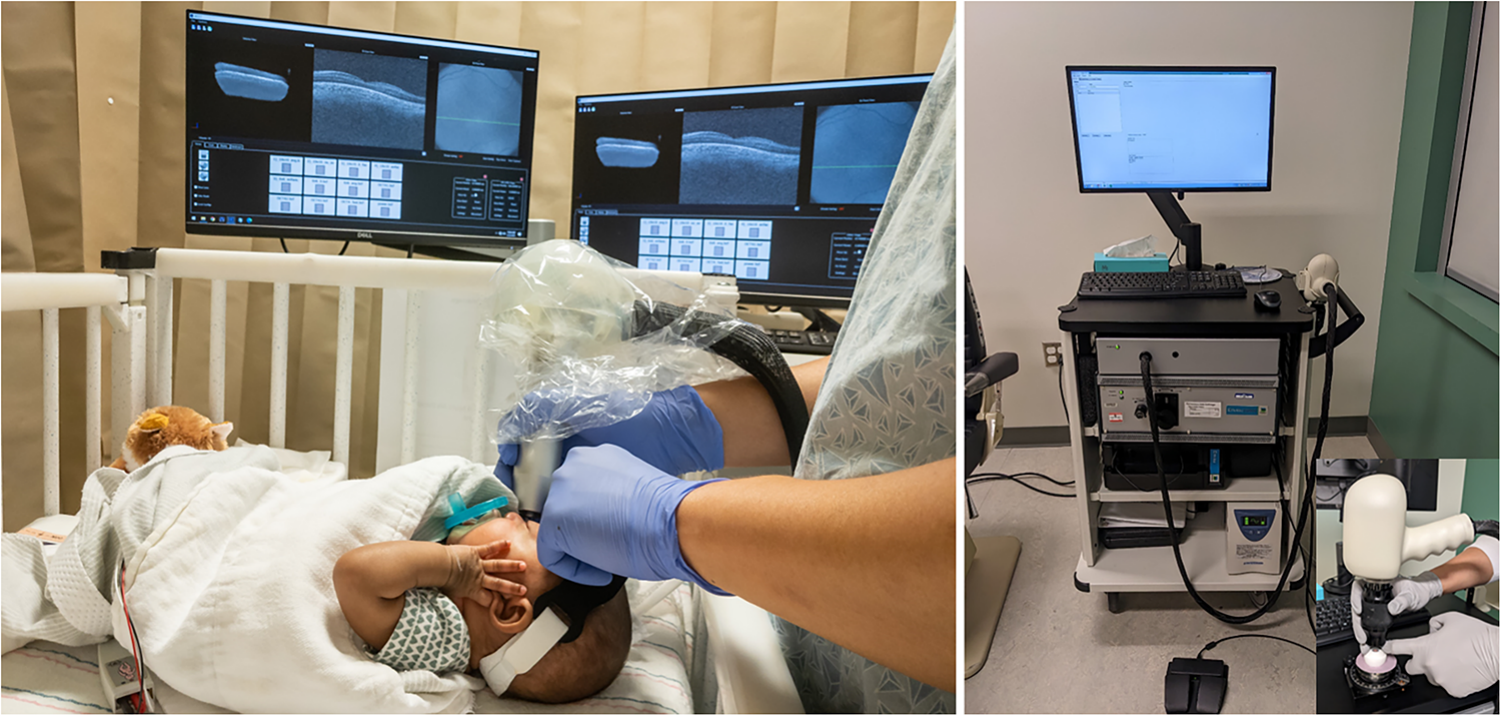
Handheld optical coherence tomography (OCT) imaging using (left) swept source investigational high-speed, non-contact OCT system in the intensive care nursery to image a preterm infant at the bedside. The screens are mirroring and show left-to-right: the three dimensional OCT volume, the selected cross-sectional OCT B-scan from the volume, and the *en face* retinal view of the scan (in which retinal vessels appear as dark lines), (Right) spectral domain commercial non-contact OCT system (Envisu 2300, Leica Microsystems) with inset showing the handheld probe being used to image a model eye.

Most OCT imaging in the ICN has been performed using handheld, non-contact OCT systems due to the portability and flexibility provided by these systems for infant imaging at the bedside. There is currently one commercially available spectral domain (SD) non-contact OCT system (Envisu 2300, Leica Microsystems). Several groups including ours have developed investigational swept-source (SS) OCT systems for bedside imaging in infant research (Figure 2) (30–32). These investigational SSOCT devices are 3–30 times faster than the commercial SDOCT system (17). The higher speed of image acquisition with the SSOCT system facilitates imaging in infants where stable fixation is a challenge. Currently, OCT imaging is not being used for routine ROP screening. The handheld commercial SDOCT system when available is utilized at the discretion of the pediatric ophthalmologist or the pediatric retina specialist for clinical decision making. The investigational SSOCT systems are being used only for research studies.

When imaging with non-contact OCT systems, infants are swaddled and the eyelids are held open gently with the imager's fingertips for imaging. An eyelid speculum is not utilized since OCT does not use a bright visible light (5, 33). Clinically stable infants are positioned horizontally across the bed for ease of access, however with handheld OCT, infants can be imaged without altering their position (23). Handheld OCT systems also allow imaging when the infants are on continuous positive airway pressure (CPAP) without the removal of the mask (23). OCT does not require pupil dilation, however, dilation facilitates peripheral retinal imaging (23). Pacifiers and sucrose are commonly used at the bedside to calm a restless infant. Handheld OCT has the benefit of being less stressful when compared to an eye exam with the indirect ophthalmoscope in preterm infants, in part due to the infrared illumination rather than bright visible light for imaging (23). While OCT imaging has been shown to be less stressful than the standard-of-care eye examination, imagers typically monitor the heart rate and oxygen saturation during OCT and either pause or stop imaging if a stressful event ensues (23).

### Using retinal layers on OCT to address disease process

**Figure 3:**
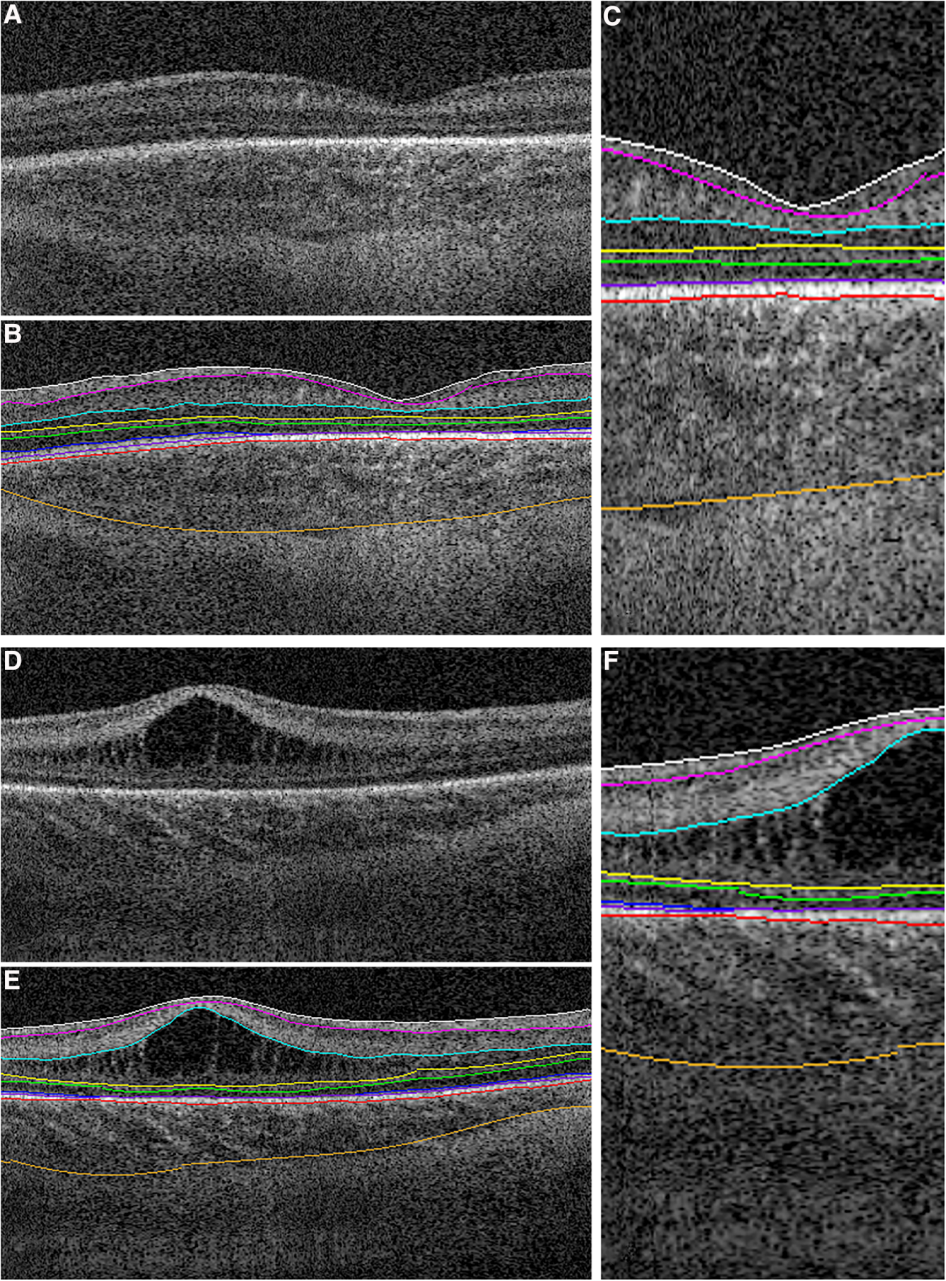
Representative foveal B-scans from macular volumes from swept-source OCT systems in infants with (**A**,**B**,**C**) no macular edema and (**D**,**E**,**F**) with macular edema. (**B**,**C**,**E**,**F**) Duke OCT Retinal Analysis Program Marking Code Baby version 2.0 semiautomated segmentation at the internal limiting membrane (white), outer borders of the nerve fiber layer (magenta), inner plexiform layer (aqua), inner nuclear layer (yellow), outer plexiform layer (green), ellipsoid zone (blue; not visualized in (**C**) and tapering at the foveal margin in (**F**)), retinal pigment epithelium (inner, purple; outer, pink), and choroid (orange). Reproduced with permission from Mangalesh et al.

On OCT images, the neurosensory retina can be divided into inner and outer layers with subdivisions described from vitreous surface to the retinal pigment epithelium: the inner composed of the internal limiting membrane (ILM), retinal nerve fiber layer (RNFL), ganglion cell layer (GCL), inner plexiform layer (IPL) and inner nuclear layer (INL), and the outer composed predominantly of photoreceptor elements within the outer plexiform layer (OPL), Henle's fiber layer at the fovea, outer nuclear layer (ONL), external limiting membrane (ELM), ellipsoid zone (EZ) and interdigitation zone (IZ) (6). Next is the retinal pigment epithelium (RPE) beneath which lies the choroid and sclera. While measurement calipers are available on most commercial OCT systems, we have developed a semiautomatic segmentation process for retinal layers to extract the desired retinal layer thickness customized for infants called the Duke OCT Retinal Analysis Program Marking Code (DOCTRAP) developed in MATLAB (Mathworks., Inc., Natick, MA) (Figure 3) (15, 34). In this review, we have included the following retinal thickness: inner retina- combined RNFL, GCL and IPL thickness, macular edema- best described quantitatively by the INL thickness, outer retina including the photoreceptors- from the OPL to the inner border of RPE, choroidal thickness- from the Bruch's membrane to the inner border of the choroidal-scleral junction and peripapillary RNFL thickness- measured at a radial distance of 1.5 mm from the center of the optic nerve in the pappilomacular bundle. We describe the findings in these layers relative to preterm infant systemic and ocular health and severity of ROP.

Normative data for each retinal layer and the peripapillary RNFL have been reported for infants and children in several studies (18, 35–39). In infants, the development of retinal layers is a complex, non-linear process that has been shown to continue after the age of 5 years and into adolescence (18). It is therefore, important to consider the age of the infant while generating thickness measurements throughout early life (5). We have reported good intergrader and intragrader reproducibility for both retinal and choroidal layer thicknesses across various age groups (15, 20, 40–42). Factor such as image quality, image tilt, variable retinal layer reflectance, imager stability for handheld OCT systems and subject body position (upright or supine) impact reproducibility across OCT systems (5, 43). To address these concerns, we evaluated the repeatability and reproducibility of handheld OCT systems compared to a tabletop system in heathy adult volunteers and reported a variation less than 3.5% for the retinal thickness at the fovea and variation less than 8.5% for peripapillary RNFL measurements for all systems (43). Infants and children may have more variability in OCT measurements due to the limitations in the ability to fixate and variable axial length through different stages of development (43).

## Fovea, inner retinal layers and pre-retinal tissue

**Figure 4:**
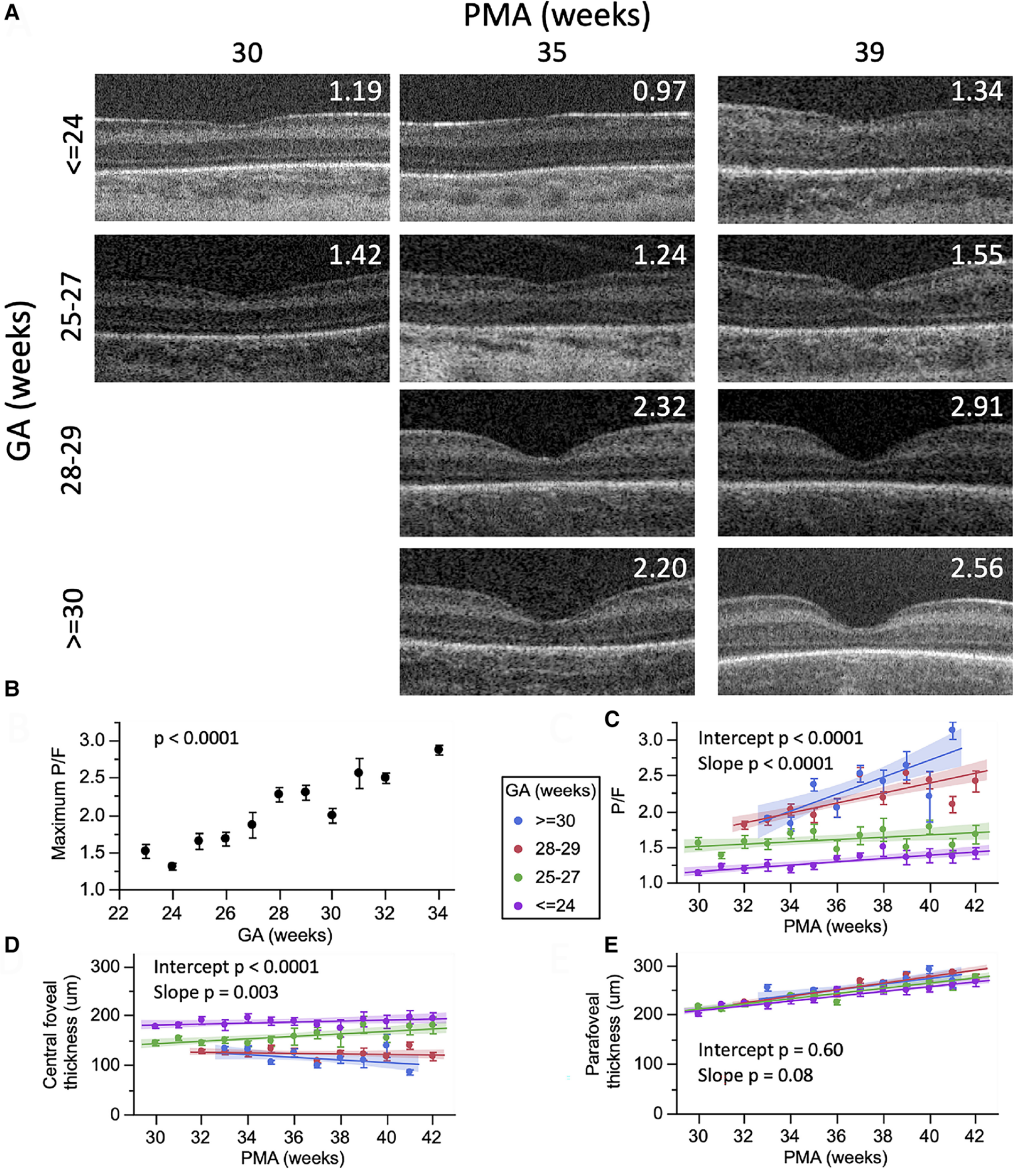
Extreme prematurity is associated with shallower foveal pits caused by thickening at the foveal center. (**A**) Example foveal OCT images from different infants born at a range of gestational ages (GA; columns) imaged across a range of PMA (rows). Inset numbers are the P/F ratio for the corresponding image. (**B**) Summary of the average maximum P/F observed per eye by GA. (**C**) Summary of average P/F across PMAs, excluding images with INL thickening. The study population was divided into GA quartiles for visualization. P/F is lower and increases less with PMA in more premature infants. (**D**) The neuroretina is thicker at the foveal center in more premature infants, and does not thin with increasing PMA. (**E**) Parafoveal neuroretinal thickness increases with PMA but is not affected by GA. Data displayed in this figure exclude images with INL thickening. *P*-values represent the results of linear mixed model analysis with gestational age treated as a continuous variable. Adapted from O'Sullivan et al.

Based on the prior OCT and histology studies in preterm infants, the inner retinal layer thickness at the foveal center decreases from the time of birth, and notable change occurs between 31 weeks to 42 weeks postmenstrual age (PMA) (6, 7, 13, 44). The process of normal and abnormal foveal development has been discussed in detail by He et al. (17). The crucial steps in the development of the fovea include- the formation of the foveal pit and the displacement of the inner retinal layers from the central fovea (6, 15, 17, 44). In BabySTEPS, at the 36 weeks PMA cross-sectional time point, we reported an association between thicker inner retina and lower gestational ages and lower birth weight (15). We further evaluated the factors affecting the inner retinal development (at the fovea) over the period of 30 weeks PMA and 42 weeks PMA and found that gestational age was the most critical factor affecting the inner retinal thickness at the fovea (Figure 4) (15). Extremely preterm infants are born with a thicker fovea and while the inner retinal thickness at the foveal center decreases due to physiological centrifugal migration of the inner retina in most infants, this process is arrested in extremely preterm infants (<28 weeks gestational age) (15). Histological studies by Hendrickson et al. (44), have shown that the foveal depression emerges between 24 and 26 weeks gestational ages indicating that majority of the inner retinal differentiation occurs before birth. In preterm infants, parturition precedes this differentiation thereby arresting foveal development (15).

The association between gestational age and ROP is well-known, hence it was not surprising to find that the inner retinal layer thickness at the fovea increased with higher ROP stages (stages 0–2) at 36 weeks. We however, did not find an association with plus disease at 36 weeks. This lack of association may have been due to fewer infants at 36 weeks with plus disease and more severe ROP (stage 3) at the time of OCT imaging (15). In an observational study, Vogel et al. longitudinally followed infants who underwent treatment for ROP and reported that eyes treated for ROP with laser photocoagulation were less likely to have all inner retinal layers present at the fovea when compared to those treated with intravitreal bevacizumab (13). While the O'Sullivan et al. study showed that foveal differentiation is potentially arrested at birth and changes little postnatally (15), this difference in the inner retinal thickness at the fovea after treatment for ROP may be attributed to the difference in vascular endothelial growth factor (VEGF) expression between anti-VEGF therapies like bevacizumab vs. laser photocoagulation (13).

In addition to classic findings of immature foveal development, OCT provides unique perspectives of the developing retinal structures, vasculature and extraretinal neovascularization corresponding to the clinical stages of ROP (12, 45–47). Some distinct findings associated with ROP stages include thickening of the inner retina at the vascular-avascular junction, presence of neovascular buds arising from the inner retinal surface and preretinal neovascularization at and behind the vascular-avascular junction along with elevation and splitting of the inner retinal layers in higher stages of ROP (45, 47). Identification of ROP specific disease findings coupled with the ability to measure extraretinal neovascular disease activity has enabled more research in the regression and reactivation of ROP (48). In infants with plus disease, the tortuous retinal vessels both bulge out of the inner retinal surface and also deform the adjacent and underlying retina (12, 49). Hyperreflective spots or bands may occur within the vitreous and retina that reflect vitreous organization with traction, exudates or inflammation in higher ROP stages (50).

Other diseases such as albinism impact foveal development and visual function. In one of the earliest uses of research handheld OCT, we identified the absence of the foveal pit and persistence of inner layers in young children such as with albinism or Hermansky-Pudlak syndrome (51). Inner retina also has loss of neural tissue associated with focal ischemia due to transient retinal vascular impairment in infancy and mosaicism such as in incontinentia pigmenti, familial exudative vitreoretinopathy (FEVR) may also cause widespread thinning of the retina evident with the loss the foveal pit. Similar to ROP, fronds of preretinal neovascularization at and behind the vascular-avascular junction can be visualized on OCT in infants with FEVR.

### Significance

From OCT imaging at the bedside, and the ability to follow the process of foveal development over time, we have learnt that gestational age plays a crucial role in the process of foveal development. Extremely preterm infants are vulnerable and affected by other systemic health conditions (52) that could also independently contribute to alterations in the inner retina both during the intrauterine period and postnatally. From the studies thus far, we have learnt that severe prematurity interrupt inner retina development however, we are unsure if this may be a consequence of prematurity alone, presence and severity of ROP, systemic health or a combined effect of all these factors. Since preterm birth is associated with various ocular and systemic diseases, and the long term survival outcomes including vision is an important concern, it is pertinent to explore the association between systemic health factors especially those implicated with preterm birth such as bronchopulmonary dysplasia or necrotizing enterocolitis with the inner retina to understand if ongoing systemic injury is reflected in the inner retina. Furthermore, OCT imaging in ROP and other pediatric diseases presents unique findings that may be utilized to determine visual prognosis and response to treatment in infants and children.

## Macular edema

Macular edema in preterm infants was first identified on OCT with an incidence estimated to be between 19% and 79% depending on the population (10, 15, 53, 54). Macular edema on OCT appear as hyporeflective spaces typically found in the fovea and parafoveal region, bilateral, symmetric, isolated to the inner nuclear layer and often causes bulging and disrupt the foveal contour (Figure 3) (10). While the onset and duration of macular edema remains unknown, it has been detected as early as 30 weeks PMA (55). Unlike adult macular edema which is primarily pathological, the etiology of macular edema in preterm infants is still unclear. We observed late macular leakage in some infants with moderate to severe edema which led us to speculate that macular edema may represent immature foveal vascular development and/or a delayed formation of the blood-retinal barrier (56). Anwar et al. hypothesized that edema in infants may be due the immaturity of the ELM which forms a part of the blood retinal barrier, however they did not find a difference with either presence or absence of ELM between infants with and without edema (57). Another possible hypothesis is the swelling of Müller cells due to increased acronym expanded in the previous section. VEGF (10, 26, 57, 58) however, the association between edema and ROP severity remains controversial. Although, the etiology of edema is still unknown, we have reported an association between poor neurodevelopmental outcomes and the presence of edema (19). In 53 preterm infants with macular edema who underwent neurodevelopmental testing with Bayley Scales at 2 years, we found that presence of macular edema was associated with poor language and motor skills (19). We are currently further evaluating this association between macular edema and neurodevelopmental outcomes in the larger BabySTEPS trial. Other etiologies proposed include mechanical traction since macular edema was associated with the presence of vitreous strands (50) and supported by our finding of optic nerve head deformation likely caused by vitreous traction in FEVR (59). The association between macular edema and ROP severity is still being debated, due to reports of onset and resolution of macular edema both before and after treatment for ROP (10, 11, 15, 53, 60). Erol et al. from Turkey reported an association between macular edema and higher ROP stages (26) and Vinekar et al. reported the presence of macular edema with stage 2 ROP in Asian Indian infants (11, 58). In BabySTEPS, presence of edema did not reach statistical significance when compared to ROP stage or plus disease, however the inner nuclear layer (the layer in which the edema forms) was found to be thicker with higher stages of ROP (15). The association between macular edema and ROP treatment has also been inconclusive thus far possibly due to the heterogeneity of the populations (13, 26). We looked for macular edema in term-born infants, and in striking contrast to preterm infants, found traces of edema only very rarely, except in infants with systemic disease. There are two different kinds of edema seen in sick term infants: diffuse parafoveal edema without foveal contour disruption in infants with hypoxic ischemic encephalopathy (22), and the dramatic thickening of the retina centrally, similar to severe edema in preterm infants, in a child with liver failure due to neonatal hemochromatosis—whose edema resolved after liver transplant (9).

### Significance

Macular edema is prevalent in the preterm infant population and the impact of edema on preterm infants is yet to be determined. Exploring the associations with systemic diseases and re-evaluating the association between macular edema and neurodevelopmental outcomes has only started and may shed light on whether macular edema is a reflection of ongoing systemic or neurological disease process or simply a physiological consequence of preterm birth.

## Outer retina, photoreceptors and retinal detachment

The outer retina, which includes the OPL, ONL and remaining photoreceptor layers to the retinal pigment epithelium, increases in retinal thickness over time at the fovea in preterm infants. This increase is attributed predominantly to the process of elongation of photoreceptor outer segments, though cone packing also contributes to the thickness (6, 7, 61). Preterm infants have a more immature outer retina when compared to term infants (62). The EZ band, a hallmark of photoreceptor development was not visible in preterm infants at 36 weeks PMA (15) and appeared at around 40–42 weeks PMA (62). In BabySTEPS, we showed that severity of prematurity has an impact on the outer retinal development at the fovea (15), and Akula et al. found that severity of ROP impacted both outer retinal development and visual acuity (61). Vogel et al. showed that infants who were treated with laser photocoagulation for ROP were less likely to have the EZ band at the fovea (13). These associations suggest that photoreceptor maturation is potentially delayed after treatment for ROP thereby affecting visual acuity.

Retinal detachment and retinal schisis may be difficult to differentiate on clinical examination or photographs, and the distinction is simplest on OCT imaging, where the outer retina remains attached in schisis—while retinal layers split—whereas outer retinal layers separate from the retinal pigment epithelium in a detachment. This is important as vitreoretinal surgery indications differ for schisis vs. detachment in older children and adults and will likely be impacted by our OCT reports on these differences in preterm infants (4, 63). OCT imaging is recognized as a valuable tool to identify whether the foveal center is involved in retinal detachments in ROP (differentiating Stage 4A-macula attached—from 4B—macula detached, retinal detachment), as noted in the ICROP3 (64).

### Significance

The outer retina in preterm infants is impacted by prematurity. Outer retinal specializations have been reported to strongly correlate with visual acuity (15). The impact of systemic diseases on the outer retinal development, particularly the photoreceptors maybe worth exploring. In addition to the outer retinal development, OCT imaging demonstrates clear benefit in evaluating advanced ROP, particularly identifying foveal involvement and determining the nature and degree of retinal elevation (schisis vs. detachment). Further investigation may help determine the sensitivity, specificity and the predictive value of OCT imaging in advanced ROP which may alter future clinical practice.

## Choroid

**Figure 5:**
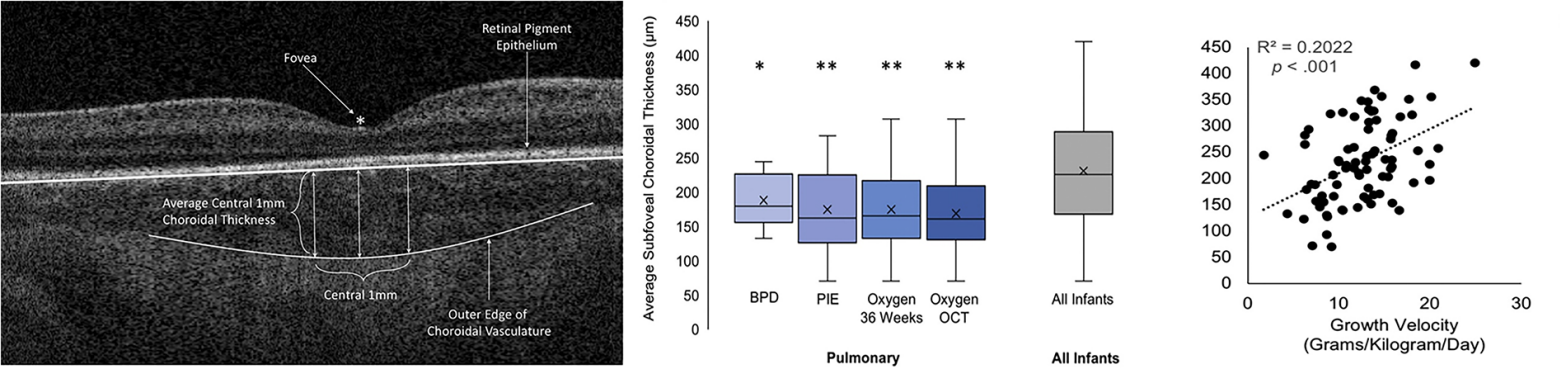
(Left) Optical coherence tomography (OCT) image showing the method for measuring the central 1 mm subfoveal choroidal thickness, (middle) Box-and-whisker plots illustrating the relationship between the presence of pulmonary findings and average 1 mm subfoveal choroidal thickness, (Right) Scatterplot representing the relationship between growth velocity and average 1 mm subfoveal choroidal thickness. BPD, bronchopulmonary dysplasia; Oxygen OCT, required oxygen supplementation at the time of OCT imaging; Oxygen 36 Weeks, required oxygen supplementation at 36 weeks’ postmenstrual age; PIE, pulmonary interstitial emphysema. Adapted with permission from Michalak S et al.

The choroid is the highly vascular layer between the retinal pigment epithelium and the sclera that provides oxygen to and removes waste products from the RPE and photoreceptors (17, 65). In infants, the choroid is visualized on OCT below the retinal pigment epithelium as hypo-and hyper-reflective areas and the choroidal thickness is typically measured from the Bruch's membrane to the choroid-scleral junction at the foveal center or across the foveal center 1 mm (15, 65). In 2013, we demonstrated that the choroid continues to develop after birth in preterm infants, however, the choroidal thickness in preterm infants lags behind that of term infants (8). Erol et al. in 2016 showed that thicker choroid was associated with a higher birth weight in preterm infants (16). Our study (Michalak et al.) and He et al. found that pulmonary status and oxygen supplementation impact choroidal thickness (66, 67). In a cross-sectional study of OCT imaging at 36 weeks PMA in 82 preterm infants in BabySTEPS (15), we evaluated the association between choroidal thickness and demographics and systemic health factors including cardiac abnormalities, hyper-inflammatory conditions and pulmonary status (66). A thinner choroid was independently associated with use of supplemental oxygen and with slower postnatal growth velocity (Figure 5) (66). Most preterm infants require some oxygen supplementation and the ideal oxygen saturation target to avoid hypoxia and hyperoxia is still under debate (68, 69). We postulated that in preterm infants receiving supplemental oxygen, increased oxidative stress and downregulation of VEGF may arrest the vascular development resulting in a thinner choroid (66). The most common systemic health factors associated with a thinner choroid were pulmonary conditions that were either directly or indirectly related to the use of supplemental oxygen such as bronchopulmonary dysplasia and pulmonary interstitial emphysema (66). He et al. also found an association between thinner choroid and higher levels of oxygen supplementation in preterm infants (67). They included gestational age, birth weight, PMA at imaging and the fraction of inspired oxygen (FiO_2_) at 30 weeks, 36 weeks and the day of OCT imaging in their analysis, where higher FiO_2_ at 30 weeks was independently associated with choroidal thinning implying that choroidal thickness was mainly impacted by early postnatal oxygen supplementation (67). Similar to our hypothesis, He et al. also concluded that hyperoxia-induced vascular attenuation may be responsible for the choroidal thinning (66, 67).

The second association of interest reported by us in Michalak et al. was that of slower growth velocity with thinner choroid in preterm infants (66). Growth velocity (grams per kilogram per day of weight gain) was assessed using the 2-point average method formula: (*W*_2_ – *W*_1_)/[*W*_2_ + *W*_1_/2]/1000/number of days, where *W*_2_ was the infant's weight on the day of OCT imaging and *W*_1_ was the infant's weight on day 7 of life (66). Growth velocity and postnatal weight gain have been increasingly associated with the risk of developing ROP in preterm infants (70, 71). While at this time, the exact mechanism by which poor postnatal weight gain impacts choroidal thickness is still unknown, studies in older former preterm and full-term infants have reported an association between lower birth weight with thinner choroid in children (72, 73). They have attributed this association to global developmental delay which is a known consequence of preterm birth (72, 73). This is also supported by evidence from our recent report from BabySTEPS, where we observed a linear growth rate of 14.8 µm/week of the choroid from 30 to 38.4 weeks and then cessation of growth, with a growth rate of 0.3 µm/week from 38.4 to 60 weeks. However, we noted that extremely low birth weight and extremely preterm infants had significantly slower initial growth rate (42).

Several studies have found a correlation between ROP and choroidal thinning (8, 15, 16, 67). In Moreno et al, we reported thinner choroid in eyes of preterm infants and in those infants with ROP (8). Erol et al. using 850 nm-centered handheld OCT reported an association between choroidal thinning with higher ROP stages (16), while in BabySTEPS, using infrared wavelength OCT that has deeper signal penetration in the choroid, we found that plus disease rather than ROP stage impacted choroidal thickness (15).

We have also identified choroidal changes in infants associated with inherited or newborn diseases such as neonatal hemochromatosis (9) and Poretti-Boltshauser syndrome (74), and that choroidal neovascularization in infants, visible on OCT, may occur from disease or at the margin of chorioretinal colobomas (75), and is an often unrecognized cause of vision loss (9, 74, 75). Other groups have also identified choroidal lesions associated with retinal effects of presumed intrauterine Zika virus infections (76, 77), deposits in the choroid such as in oxalate retinopathy (78), and other chorioretinal lesions (79). In all of these conditions, the depth and pattern of choroidal involvement is not possible without OCT imaging.

### Significance

While the exact mechanism underlying the complex relationship between oxygen supplementation, choroidal thinning and ROP is still unknown, it is pertinent to recognize that these published studies demonstrate associations and not causality (66, 67). Future directions include additional studies and assessment of the long-term implication of oxygen supplementation and poor postnatal growth related choroidal thinning on visual function and neurodevelopmental outcomes in preterm infants. The depth of visualization, especially of the choroid has led to the identification of sub-clinical findings in diseases which may be useful in the development of newer disease classifications and facilitate objective assessment of the choroid over time and following treatment.

## Optic nerve and retinal nerve fiber layer

A multitude of diseases can affect the infant brain, optic nerve and retina and are conventionally recognized from swelling of or atrophy and pallor of the optic nerve head, as viewed with ophthalmoscopy or photographs. The optic nerve contains unmyelinated retinal ganglion cell axons that constitute the innermost layer of the neurosensory retina and converge here to exit the eye. These fibers remain unmyelinated until they cross the lamina cribrosa forming the optic nerve, at which point they are surrounded by myelin of the oligodendrocytes (or by pre-oligodendrocytes after very preterm birth) and they project through the optic chiasm into the optic tract and pass near the third ventricle before they reach the lateral geniculate nucleus, the midbrain pretectal area, the suprachiasmatic nucleus or the superior colliculus (17, 80). OCT imaging of the optic nerve head and surrounding retinal nerve fiber layer (RNFL) provide more nuanced measures of repeatable and accurate thickening and thinning, captures disease changes not readily visible to the clinician, and can be used to measure and thus monitor disease and treatment effects over time. This is especially important for infants who cannot report symptoms of visual field loss from disease.

As with the brain, the ganglion cell axons are developing during the preterm period and it is challenging to differentiate disease effects from the age-appropriate preterm optic nerve appearance. In an early study with Tong et al. (81), we have compared the optic nerve head parameters between preterm and term infants using OCT and reported a larger cup-to-disc ratio in preterm infants, however, within the preterm infant cohort we found that infants with a diagnosis of periventricular leukomalacia had a larger cup-to-disc ratio and that infants with a larger cup-to-disc ratio had worse cognitive skills when assessed over 18 months later by Bayley scales. As has been found in adult optic nerve disease, we found that RNFL thickness was a more reliable measure of the retinal ganglion cell integrity when compared to optic nerve head parameters (35, 81).

**Figure 6:**
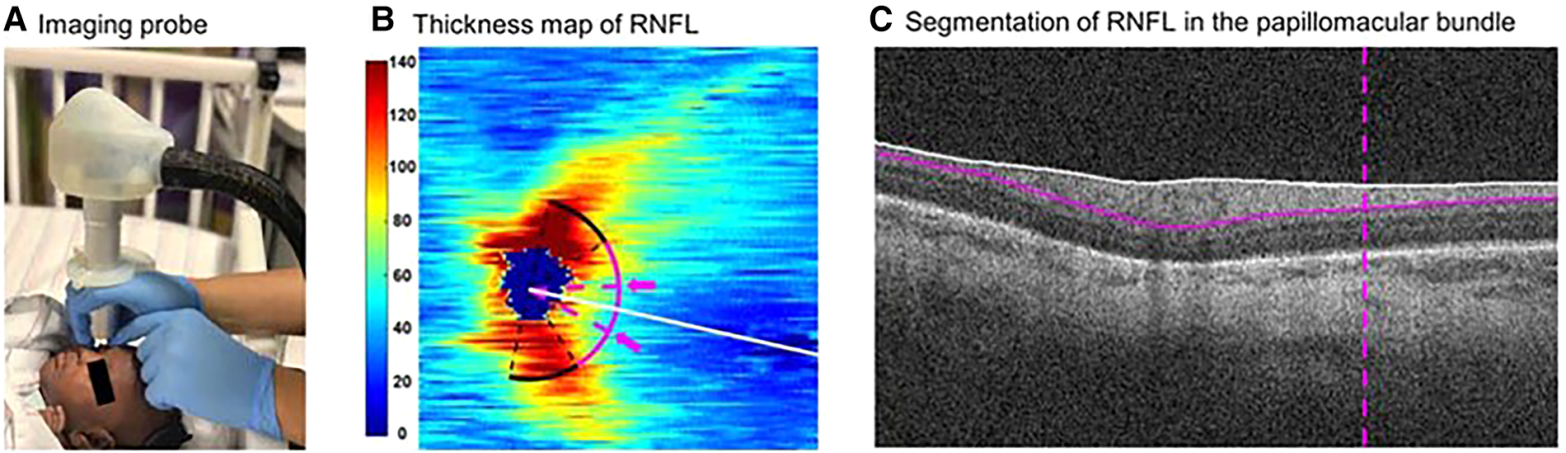
Demonstration of the optical coherence tomography imaging probe and segmentation of retinal nerve fiber layer (RNFL). (**A**) The ultracompact, non-contact, handheld imaging probe being used to image an infant at the bedside in the Duke intensive care nursery. (**B**) Thickness map (in µm) of peripapillary RNFL derived from swept-source optical coherence tomography volumes of an eye of a preterm infant in our cohort. The white line represents the organizing axis from the optic nerve center to the fovea. The pink arc represents both temporal quadrants (arc from −45 to +45 degrees relative to the organizing axis) at 1.5 mm from the optic nerve head center. The arc between 2 dashed pink lines and arrows represents the papillomacular bundle (arc from −15 to +15 degrees relative to the organizing axis). (**C**) Segmentation of RNFL (between the white and pink solid lines) in the papillomacular bundle (vertical dashed pink line) in an optical coherence tomography b-scan of the same eye. Reproduced with permission from Shen LL et al.

**Figure 7:**
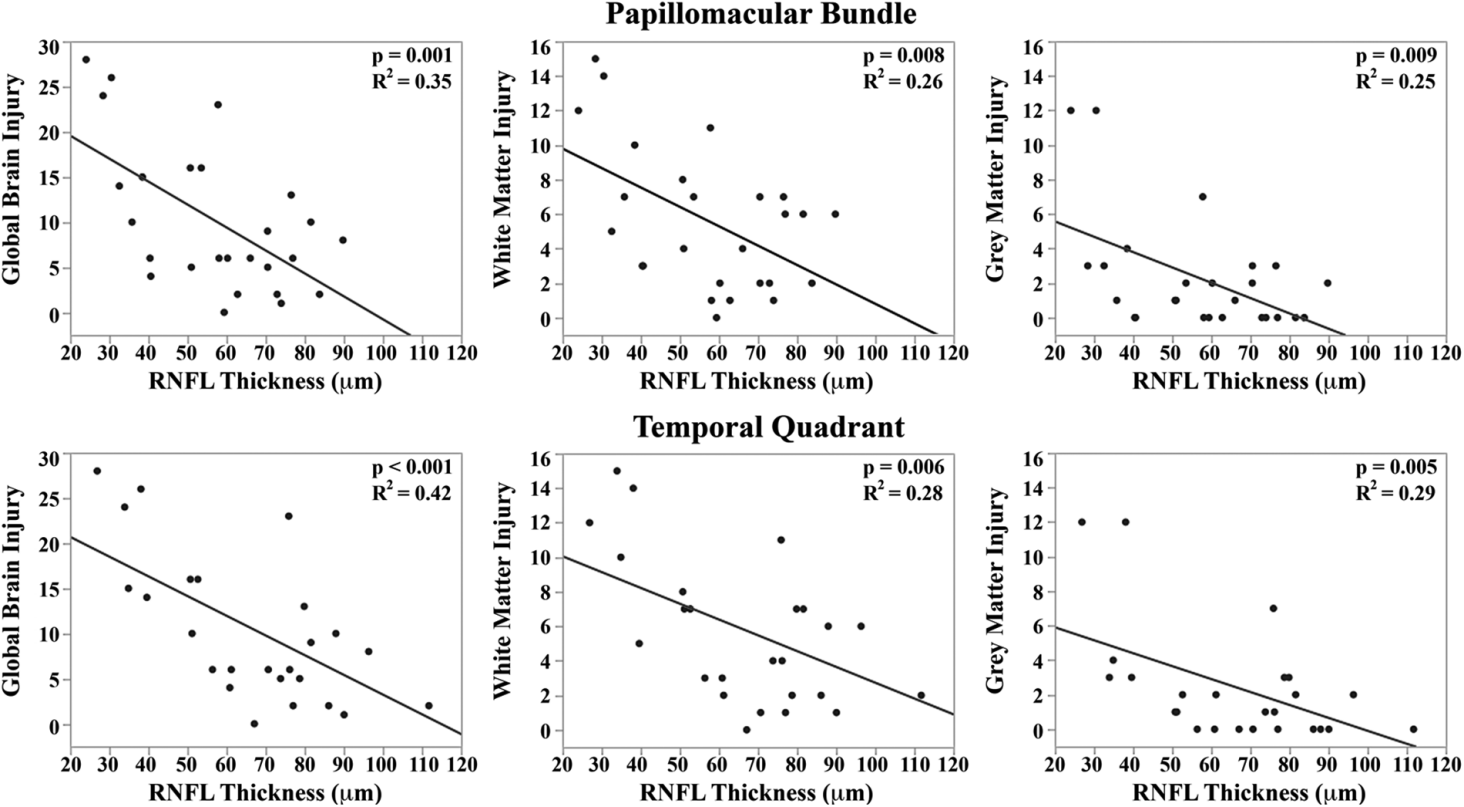
Correlation between mean retinal nerve fiber layer (RNFL) thickness along the papillomacular bundle **(Top)** and temporal quadrant **(bottom)** and global brain magnetic resonance imaging (MRI) lesion burden index, white matter injury, and gray matter injury for 26 very preterm infants who underwent brain MRI while in the intensive care nursery. Thinner RNFL across either arc (papillo-macular bundle or temporal quadrant) correlated with an increase in global brain injury, white matter injury, and gray matter injury. Reproduced with permission from Rothman et al.

Hence, OCT imaging of the RNFL presents a unique opportunity to non-invasively evaluate the central nervous system, and OCT-based RNFL thickness has been a non-invasive biomarker for axonal injury, independent of myelination changes and repeatedly accessible at the bedside (Figure 6) (40, 82). In preterm and term infants, we have demonstrated and validated the utility and reproducibility of measuring RNFL thickness at a radial distance of 1.5 mm from the center of the optic nerve in the sector between the optic nerve and macula (20, 35, 40). In a study of older infants and young children, we demonstrated the stability of RNFL measurement from 6 months to 5 years of age (38). In an early study in a cohort of 57 very preterm infants, we evaluated the relationship between RNFL thickness and common preterm infant pathologies such as hydrocephalus, intraventricular hemorrhage, bronchopulmonary dysplasia and sepsis/necrotizing enterocolitis (NEC) (20). We noted a trend towards thinner RNFL in preterm infants with sepsis/NEC and a significant relationship between RNFL thickness and brain abnormalities on magnetic resonance imaging (MRI) using a modified Kidokoro score used to assess the global brain lesion burden index which comprised of white matter, gray matter and cerebellar subscores (Figure 7) (20). We also found a significant correlation between thinner RNFL and worse cognitive and motor skills as assessed by the Bayley Scales (20). This study highlighted that RNFL thickness may be a promising non-invasive, bedside biomarker of brain injury and subsequent neurodevelopmental outcome as an adjunct tool to clinical MRI and is currently being evaluated in the larger BabySTEPS trial (20, 82).

From the subsequent prospective BabySTEPS, we first assessed the impact of birth, demographic factors and ROP on RNFL thickness at 36 weeks PMA in very and extremely preterm and very and extremely low birth weight infants who are vulnerable for neurodevelopmental and visual impairments (40, 83, 84). While extremely preterm infants and extremely low birth weight preterm infants had significantly thinner RNFL when compared to very preterm infants and very low birth weight infants respectively, birth weight stood out as the only independent factor influencing RNFL thickness, with an estimated 5.2 µm increase in RNFL thickness with every 250 g increase in birth weight (40). It appears that antenatal events such as fetal malnutrition can disturb cellular growth and differentiation in the central nervous system and ganglion cells of the eye (40). Supporting this hypothesis, Garcia-Filion et al. found a much higher prevalence of low maternal pregnancy weight gain among infants with optic nerve hypoplasia (85) and Lenzi et al. in an animal study, demonstrated that a high protein and lipid diet in the prenatal and postnatal period in rats had a favorable influence on optic nerve development (86).

Subsequently in the same BabySTEPS cohort, we investigated the association between 29 systemic health factors and RNFL thickness (87). We found that low infant weight at the time of OCT imaging (36 weeks PMA) and sepsis/NEC were independently associated with thinner RNFL suggesting that poor intrauterine and postnatal growth as well as systemic inflammatory conditions may adversely impact optic nerve development in preterm infants. We suggest two possible mechanisms explaining the association between thinner RNFL and sepsis/NEC: a direct effect of systemic inflammatory mediators may disrupt the blood-retinal barrier and damage the retinal ganglion cells or axons (88, 89), or systemic inflammation may impact regions of the visual track within the brain, in turn, causing retrograde trans-synaptic degeneration of retinal ganglion cells (90). The latter theory is consistent with the Shah et al. report of significant white matter injury in infants with sepsis/NEC (91).

RNFL thickness in preterm infants follows a biphasic pattern of change between 30 and 60 weeks PMA. In BabySTEPS, we found that the RNFL thickness increases at 1.8 µm/week from 30 to 37.8 weeks PMA and then decreases at −0.3 µm/week from 37.8 to weeks PMA (41). We did not find an association between RNFL thickness and macular edema in either BabySTEPS or our earlier report with Rothman et al. (20, 41). This understanding of the biphasic thickness change may be useful when evaluating the longitudinal impact of diseases such as ROP and interventions such as ROP treatment on infant retinal development.

To further study of effects of ischemia on the ganglion cells and axons of neonates, we have performed research bedside OCT imaging in those in cooling protocols and suspected of having hypoxic ischemic encephalopathy. The imaging was performed without pupil dilation as the infrared light of OCT does not constrict the pupil. We demonstrated an association between RNFL thinning and hypoxic injury, with profound loss of ganglion cell layer in some infants. Because thinning was already present within days of birth in several infants, we postulated that retinal injury evident as thinning may be due to the direct impact of acute or chronic hypoxia on the RNFL, or due to the process of retrograde injury (22). To examine other types of tractional forces acting on the optic nerve head, we imaged infants with familial exudative vitreoretinopathy, a vascular disease similar to ROP but in term-born infants, with OCT we could identify progressive deformation elevating the inner surface of the optic nerve head; this was well before any elevation was apparent to the clinician (59). We have also demonstrated visualization of papilledema and RNFL loss in a preterm born child with obstructive hydrocephalus (33). More recently, investigators have suggested that OCT measures of the RNFL and retina may be a potentially sensitive indicator of elevated intracranial pressure in infants and young children such as with craniosynostosis (92, 93); this remains to be validated, and we are not aware of reports for the preterm population. Others have shown that OCT imaging of RNFL thickness in infants and young children enables earlier identification of damage from optic nerve glioma, with ongoing validation studies (94–96). OCT has also been utilized to assess optic nerve injury in infants with primary congenital glaucoma or glaucoma after infant cataract surgery (97), and the OCT imaging has revealed previously unrecognized injury to other retinal areas, presumed to be ischemic in nature. Handheld OCT imaging is also currently under evaluation for assessment of early vigabatrin effects on the retina in infants treated for infantile spasm. This points to potential future utility for OCT in monitoring for similar high risk off target therapeutic effects in preterm infants (98).

### Significance

RNFL is an extension of the diencephalon of the central nervous system. Research thus far demonstrates that birth weight, systemic inflammation such as sepsis/NEC and brain injury have an impact on the RNFL thickness. With the high incidence of poor neurodevelopmental outcomes in extremely preterm infants (83), RNFL could serve as a potential early, repeatable non-invasive biomarker for neurodevelopment (82). Longitudinal, prospective studies are needed to validate these findings and identify the role of RNFL thickness in clinical care. In addition to preterm infant systemic health, RNFL thickness has shown promise in a myriad of other pediatric diseases such as hydrocephalus and optic nerve gliomas as a surrogate marker of pregeniculate visual pathway integrity, and may provide essential information required to make treatment decisions, especially since visual acuity testing in these infants and toddlers may be challenging. The ability to longitudinally assess RNFL thickness is also beneficial to detect structural retinal biomarkers for either therapeutic or toxic effects of drug therapies.

## Future directions

Advances in OCT imaging in preterm infants have added to understanding of retina development, however, given the complexity and overlap of gestational age, birthweight, systemic health, hypoxic injury and hyperoxia with ROP, we have yet to determine and isolate retinal biomarkers for systemic health from those related to ROP. In studying potential biomarkers within the eye, it is exceedingly important to understand the progressive and rapid changes of development of retinal and choroidal layers in preterm infants, and the complex interactions between systemic health, development and ROP. Fortunately, through bedside OCT imaging in infants, we have begun the process of measuring retinal layers and identifying OCT findings and their associations with ocular and systemic disease. Thus far, the developmental data across this population has provided a reference framework for future clinical investigations into the early onset and pathways of abnormal development and for therapeutic interventions for diseases or injury affecting visual outcomes. Additionally, these studies have also provided an objective basis for the design of follow up studies like BabySTEPS 2 (ClinTrials.gov NCT04995341) that aim to address not only the preservation of vision in preterm infants but also neonatal care associated with better systemic and neurodevelopmental outcomes. As with adults, we expect that OCT imaging of infants will not be restricted for one disease, and that imaging will prove useful across a spectrum of genetic and developmental conditions in the future.

## Ethics statement

Written informed consent was obtained from the minor's legal guardian/next of kin for the publication of any potentially identifiable images or data included in this article.

## References

[1] Joshi MM Trese MT Capone A Jr. Optical coherence tomography findings in stage 4A retinopathy of prematurity: a theory for visual variability. Ophthalmology. (2006);113(4):657–60. 10.1016/j.ophtha.2006.01.007 16581425

[2] Vinekar A Sivakumar M Shetty R Mahendradas P Krishnan N Mallipatna A A novel technique using spectral-domain optical coherence tomography (spectralis, SD-OCT+HRA) to image supine non-anaesthetized infants: utility demonstrated in aggressive posterior retinopathy of prematurity. Eye (Lond). (2010) 24(2):379–82. 10.1038/eye.2009.313 20057510

[3] Scott AW Farsiu S Enyedi LB Wallace DK Toth CA. Imaging the infant retina with a hand-held spectral-domain optical coherence tomography device. Am J Ophthalmol. (2009) 147(2):364–73.e2. 10.1016/j.ajo.2008.08.010 18848317

[4] Chavala SH Farsiu S Maldonado R Wallace DK Freedman SF Toth CA. Insights into advanced retinopathy of prematurity using handheld spectral domain optical coherence tomography imaging. Ophthalmology. (2009) 116(12):2448–56. 10.1016/j.ophtha.2009.06.003 19766317 PMC3514074

[5] Maldonado RS Izatt JA Sarin N Wallace DK Freedman S Cotten CM Optimizing hand-held spectral domain optical coherence tomography imaging for neonates, infants, and children. Invest Ophthalmol Visual Sci. (2010) 51(5):2678–85. 10.1167/iovs.09-4403 20071674 PMC2868489

[6] Maldonado RS O'Connell RV Sarin N Freedman SF Wallace DK Cotten CM Dynamics of human foveal development after premature birth. Ophthalmology. (2011) 118(12):2315–25. 10.1016/j.ophtha.2011.05.028 21940051 PMC3496560

[7] Vajzovic L Hendrickson AE O'Connell RV Clark LA Tran-Viet D Possin D Maturation of the human fovea: correlation of spectral-domain optical coherence tomography findings with histology. Am J Ophthalmol. (2012) 154(5):779–89.e2. 10.1016/j.ajo.2012.05.004 22898189 PMC3612897

[8] Moreno TA O'Connell RV Chiu SJ Farsiu S Cabrera MT Maldonado RS Choroid development and feasibility of choroidal imaging in the preterm and term infants utilizing SD-OCT. Invest Ophthalmol Visual Sci. (2013) 54(6):4140–7. 10.1167/iovs.12-11471 23652488 PMC3684216

[9] Maldonado RS Freedman SF Cotten CM Ferranti JM Toth CA. Reversible retinal edema in an infant with neonatal hemochromatosis and liver failure. J AAPOS. (2011) 15(1):91–3. 10.1016/j.jaapos.2010.11.016 21397814 PMC3059895

[10] Maldonado RS O'Connell R Ascher SB Sarin N Freedman SF Wallace DK Spectral-domain optical coherence tomographic assessment of severity of cystoid macular edema in retinopathy of prematurity. Arch Ophthalmol. (2012) 130(5):569–78. 10.1001/archopthalmol.2011.1846 22232366 PMC3515869

[11] Vinekar A Avadhani K Sivakumar M Mahendradas P Kurian M Braganza S Understanding clinically undetected macular changes in early retinopathy of prematurity on spectral domain optical coherence tomography. Invest Ophthalmol Visual Sci. (2011) 52(8):5183–8. 10.1167/iovs.10-7155 21551410

[12] Maldonado RS Yuan E Tran-Viet D Rothman AL Tong AY Wallace DK Three-dimensional assessment of vascular and perivascular characteristics in subjects with retinopathy of prematurity. Ophthalmology. (2014) 121(6):1289–96. 10.1016/j.ophtha.2013.12.004 24461542 PMC4070381

[13] Vogel RN Strampe M Fagbemi OE Visotcky A Tarima S Carroll J Foveal development in infants treated with bevacizumab or laser photocoagulation for retinopathy of prematurity. Ophthalmology. (2018) 125(3):444–52. 10.1016/j.ophtha.2017.09.020 29103792

[14] Vinekar A Mangalesh S Jayadev C Maldonado RS Bauer N Toth CA. Retinal imaging of infants on spectral domain optical coherence tomography. BioMed Res Int. (2015) 2015:782420. 10.1155/2015/782420 26221606 PMC4506845

[15] O’Sullivan ML Ying GS Mangalesh S Tai V Divecha HR Winter KP Foveal differentiation and inner retinal displacement are arrested in extremely premature infants. Invest Ophthalmol Visual Sci. (2021) 62(2):25. 10.1167/iovs.62.2.25 PMC7900865 33599735

[16] Erol MK Coban DT Ozdemir O Dogan B Tunay ZO Bulut M. Choroidal thickness in infants with retinopathy of prematurity. Retina. (2016) 36(6):1191–8. 10.1097/IAE.0000000000000866 26583308

[17] He Y Chen X Tsui I Vajzovic L Sadda SR. Insights into the developing fovea revealed by imaging. Prog Retinal Eye Res. (2022) 90:101067. 10.1016/j.preteyeres.2022.101067 PMC12183701 35595637

[18] Lee H Purohit R Patel A Papageorgiou E Sheth V Maconachie G In vivo foveal development using optical coherence tomography. Invest Ophthalmol Visual Sci. (2015) 56(8):4537–45. 10.1167/iovs.15-16542 26200492

[19] Rothman AL Tran-Viet D Gustafson KE Goldstein RF Maguire MG Tai V Poorer neurodevelopmental outcomes associated with cystoid macular edema identified in preterm infants in the intensive care nursery. Ophthalmology. (2015) 122(3):610–9. 10.1016/j.ophtha.2014.09.022 25439600 PMC4339440

[20] Rothman AL Sevilla MB Mangalesh S Gustafson KE Edwards L Cotten CM Thinner retinal nerve fiber layer in very preterm versus term infants and relationship to brain anatomy and neurodevelopment. Am J Ophthalmol. (2015) 160(6):1296–308.e2. 10.1016/j.ajo.2015.09.015 26386157 PMC4651865

[21] Seely KR Mangalesh S Shen LL McGeehan B Ying GS Sarin N Association between retinal microanatomy in preterm infants and 9-month visual acuity. JAMA Ophthalmol. (2022) 140(7):699–706. 10.1001/jamaophthalmol.2022.1643 35653144 PMC9164120

[22] Mangalesh S Tran-Viet D Pizoli C Tai V El-Dairi MA Chen X Subclinical retinal versus brain findings in infants with hypoxic ischemic encephalopathy. Graefes Arch Clin Exp Ophthalmol. (2020) 258(9):2039–49. 10.1007/s00417-020-04738-0 32472201 PMC7442701

[23] Mangalesh S Sarin N McGeehan B Prakalapakorn SG Tran-Viet D Cotten CM Preterm infant stress during handheld optical coherence tomography vs binocular indirect ophthalmoscopy examination for retinopathy of prematurity. JAMA Ophthalmol. (2021) 139(5):567–74. 10.1001/jamaophthalmol.2021.0377 33792625 PMC8017483

[24] Smith GC Gutovich J Smyser C Pineda R Newnham C Tjoeng TH Neonatal intensive care unit stress is associated with brain development in preterm infants. Ann Neurol. (2011) 70(4):541–9. 10.1002/ana.22545 21976396 PMC4627473

[25] Nist MD Harrison TM Steward DK. The biological embedding of neonatal stress exposure: a conceptual model describing the mechanisms of stress-induced neurodevelopmental impairment in preterm infants. Res Nurs Health. (2019) 42(1):61–71. 10.1002/nur.21923 30499161 PMC6749822

[26] Erol MK Ozdemir O Turgut Coban D Bilgin AB Dogan B Sogutlu Sari E Macular findings obtained by spectral domain optical coherence tomography in retinopathy of prematurity. J Ophthalmol. (2014) 2014:468653. 10.1155/2014/468653 25544895 PMC4269090

[27] Kubsad D Ohan MA Wu JG Cabrera MT. Vitreoretinal biomarkers of retinopathy of prematurity using handheld optical coherence tomography: a review. *Front Pediatr*. (2023) 11:1191174. 10.3389/fped.2023.1191174 37325353 PMC10264674

[28] Thomas AS Hsu ST House RJ Finn AP Kelly MP Toth CA Microvascular features of treated retinoblastoma tumors in children assessed using OCTA. Ophthalmic Surg Lasers Imaging Retina. (2019) 51(1):43–9. 10.3928/23258160-20191211-06 31935302 PMC8896567

[29] Mangalesh S Chen X Tran-Viet D Viehland C Freedman SF Toth CA. Assessment of the retinal structure in children with incontinentia pigmenti. Retina. (2017) 37(8):1568–74. 10.1097/IAE.0000000000001395 28085775 PMC5776702

[30] Ni S Wei X Ng R Ostmo S Chiang MF Huang D High-speed and widefield handheld swept-source OCT angiography with a VCSEL light source. Biomed Opt Express. (2021) 12(6):3553–70. 10.1364/BOE.425411 34221678 PMC8221946

[31] Moshiri Y Legocki AT Zhou K Cabrera MT Rezaei KA Tarczy-Hornoch K Handheld swept-source optical coherence tomography with angiography in awake premature neonates. Quant Imaging Med Surg. (2019) 9(9):1495–502. 10.21037/qims.2019.09.01 31667136 PMC6785511

[32] Viehland C Chen X Tran-Viet D Jackson-Atogi M Ortiz P Waterman G Ergonomic handheld OCT angiography probe optimized for pediatric and supine imaging. Biomed Opt Express. (2019) 10(5):2623–38. 10.1364/BOE.10.002623 31143506 PMC6524583

[33] Tran-Viet D Wong BM Mangalesh S Maldonado R Cotten CM Toth CA. Handheld spectral domain optical coherence tomography imaging through the undilated pupil in infants born preterm or with hypoxic injury or hydrocephalus. Retina. (2018) 38(8):1588–94. 10.1097/IAE.0000000000001735 28570486 PMC5708150

[34] Chiu SJ Li XT Nicholas P Toth CA Izatt JA Farsiu S. Automatic segmentation of seven retinal layers in SDOCT images congruent with expert manual segmentation. Opt Express. (2010) 18(18):19413–28. 10.1364/OE.18.019413 20940837 PMC3408910

[35] Rothman AL Sevilla MB Freedman SF Tong AY Tai V Tran-Viet D Assessment of retinal nerve fiber layer thickness in healthy, full-term neonates. Am J Ophthalmol. (2015) 159(4):803–11. 10.1016/j.ajo.2015.01.017 25634528 PMC4570498

[36] Yanni SE Wang J Cheng CS Locke KI Wen Y Birch DG Normative reference ranges for the retinal nerve fiber layer, macula, and retinal layer thicknesses in children. Am J Ophthalmol. (2013) 155(2):354–60.e1. 10.1016/j.ajo.2012.08.010 23127751 PMC3545013

[37] Turk A Ceylan OM Arici C Keskin S Erdurman C Durukan AH Evaluation of the nerve fiber layer and macula in the eyes of healthy children using spectral-domain optical coherence tomography. Am J Ophthalmol. (2012) 153(3):552–9.e1. 10.1016/j.ajo.2011.08.026 22019223

[38] Lim ME Jiramongkolchai K Xu L Freedman SF Tai V Toth CA Handheld optical coherence tomography normative inner retinal layer measurements for children <5 years of age. Am J Ophthalmol. (2019) 207:232–9. 10.1016/j.ajo.2019.06.015 31229465

[39] Rotruck JC House RJ Freedman SF Kelly MP Enyedi LB Prakalapakorn SG Optical coherence tomography normative peripapillary retinal nerve fiber layer and macular data in children 0–5 years of age. Am J Ophthalmol. (2019) 208:323–30. 10.1016/j.ajo.2019.06.025 31271744

[40] Shen LL Mangalesh S McGeehan B Tai V Sarin N El-Dairi MA Birth weight is a significant predictor of retinal nerve fiber layer thickness at 36 weeks postmenstrual age in preterm infants. Am J Ophthalmol. (2021) 222:41–53. 10.1016/j.ajo.2020.08.043 32891695 PMC7930155

[41] Shen LL Mangalesh S McGeehan B Biphasic change in retinal nerve fibre layer thickness from 30 to 60 weeks postmenstrual age in preterm infants. Br J Ophthalmol. (2022). 10.1136/bjo-2022-321621 PMC10270321 36113954

[42] Michalak SM Mangalesh S Chen Y Shen LL Tai V Winter K Longitudinal choroidal development in preterm infants. Ophthalmol Sci. (2023):100359. 10.1016/j.xops.2023.100359 PMC10591002 37877004

[43] Chen X Tai V McGeehan B Ying GS Viehland C Imperio R Repeatability and reproducibility of axial and lateral measurements on handheld optical coherence tomography systems compared with tabletop system. Transl Vis Sci Technol. (2020) 9(11):25. 10.1167/tvst.9.11.25 PMC7585396 33150050

[44] Hendrickson A Possin D Vajzovic L Toth CA. Histologic development of the human fovea from midgestation to maturity. Am J Ophthalmol. (2012) 154(5):767–78.e2. 10.1016/j.ajo.2012.05.007 22935600 PMC3509500

[45] Chen X Mangalesh S Dandridge A Tran-Viet D Wallace DK Freedman SF Spectral-domain OCT findings of retinal vascular-avascular junction in infants with retinopathy of prematurity. Ophthalmol Retina. (2018) 2(9):963–71. 10.1016/j.oret.2018.02.001 30506013 PMC6261282

[46] Mangalesh S Bleicher ID Chen X Viehland C LaRocca F Izatt JA Three-dimensional pattern of extraretinal neovascular development in retinopathy of prematurity. Graefes Arch Clin Exp Ophthalmol. (2019) 257(4):677–88. 10.1007/s00417-019-04274-6 30790072 PMC6698900

[47] Nguyen TP Ni S Ostmo S Rajagopalan A Coyner AS Woodward M Association of optical coherence tomography-measured fibrovascular ridge thickness and clinical disease stage in retinopathy of prematurity. JAMA Ophthalmol. (2022) 140(11):1121–7. 10.1001/jamaophthalmol.2022.4173 36227622 PMC9562098

[48] Smith LEH Hellström A Stahl A Fielder A Chambers W Moseley J Development of a retinopathy of prematurity activity scale and clinical outcome measures for use in clinical trials. JAMA Ophthalmol. (2019) 137(3):305–11. 10.1001/jamaophthalmol.2018.5984 30543348 PMC6565513

[49] Mangalesh S Seely KR Tran-Viet D Tai V Chen X Prakalapakorn SG Integrated visualization highlighting retinal changes in retinopathy of prematurity from 3-dimensional optical coherence tomography data. JAMA Ophthalmol. (2022) 140(7):725–9. 10.1001/jamaophthalmol.2022.1344 35616956 PMC9136675

[50] Zepeda EM Shariff A Gillette TB Grant L Ding L Tarczy-Hornoch K Vitreous bands identified by handheld spectral-domain optical coherence tomography among premature infants. JAMA Ophthalmol. (2018) 136(7):753–8. 10.1001/jamaophthalmol.2018.1509 29799932 PMC6136047

[51] Chong GT Farsiu S Freedman SF Sarin N Koreishi AF Izatt JA Abnormal foveal morphology in ocular albinism imaged with spectral-domain optical coherence tomography. Arch Ophthalmol. (2009) 127(1):37–44. 10.1001/archophthalmol.2008.550 19139336

[52] Stoll BJ Hansen NI Bell EF Shankaran S Laptook AR Walsh MC Neonatal outcomes of extremely preterm infants from the NICHD neonatal research network. Pediatrics. (2010) 126(3):443–56. 10.1542/peds.2009-2959 20732945 PMC2982806

[53] Dubis AM Subramaniam CD Godara P Carroll J Costakos DM. Subclinical macular findings in infants screened for retinopathy of prematurity with spectral-domain optical coherence tomography. Ophthalmology. (2013) 120(8):1665–71. 10.1016/j.ophtha.2013.01.028 23672969 PMC3737379

[54] Vinekar A Avadhani K Sivakumar M Mahendradas P Kurian M Braganza S Macular edema in premature infants. Ophthalmology. (2012) 119(6):1288–9.e1; author reply 1289–1290.e1281. 10.1016/j.ophtha.2012.03.029 22656902

[55] Mangalesh S Wong BM Chen X Tran-Viet D Stinnett SS Sarin N Morphological characteristics of early- versus late-onset macular edema in preterm infants. J AAPOS. (2020) 24(5):303–6. 10.1016/j.jaapos.2020.06.006 32942022 PMC8006576

[56] Chen X Mangalesh S Tran-Viet D Freedman SF Vajzovic L Toth CA. Fluorescein angiographic characteristics of macular edema during infancy. JAMA Ophthalmol. (2018) 136(5):538–42. 10.1001/jamaophthalmol.2018.0467 29621379 PMC6145655

[57] Anwar S Nath M Gottlob I Proudlock FA. Severity of cystoid macular oedema in preterm infants observed using hand-held spectral domain optical coherence tomography improves weekly with postmenstrual age. Eye (Lond). (2023). 10.1038/s41433-023-02461-8 [Epub ahead of print]. 36928228 PMC10516860

[58] Vinekar A Mangalesh S Jayadev C Bauer N Munusamy S Kemmanu V Macular edema in Asian Indian premature infants with retinopathy of prematurity: impact on visual acuity and refractive status after 1-year. Indian J Ophthalmol. (2015) 63(5):432–7. 10.4103/0301-4738.159879 26139806 PMC4501141

[59] Lee J El-Dairi MA Tran-Viet D Mangalesh S Dandridge A Jiramongkolchai K Longitudinal changes in the optic nerve head and retina over time in very young children with familial exudative vitreoretinopathy. Retina. (2019) 39(1):98–110. 10.1097/IAE.0000000000001930 29190238 PMC5963956

[60] Mangalesh S Chen X Tran-viet D Sarin N Winter K Prakalapakorn SG Short-term treatment effects on retinal microanatomy in preterm infants with severe retinopathy of prematurity. Invest Ophthalmol Visual Sci. (2022) 63(7):4192-F0252.

[61] Akula JD Arellano IA Swanson EA Favazza TL Bowe TS Munro RJ The fovea in retinopathy of prematurity. Invest Ophthalmol Visual Sci. (2020) 61(11):28. 10.1167/iovs.61.11.28 PMC7500148 32936301

[62] Vajzovic L Rothman AL Tran-Viet D Cabrera MT Freedman SF Toth CA. Delay in retinal photoreceptor development in very preterm compared to term infants. Invest Ophthalmol Visual Sci. (2015) 56(2):908–13. 10.1167/iovs.14-16021 25587063 PMC4321398

[63] Chen X Prakalapakorn SG Freedman SF Vajzovic L Toth CA. Differentiating retinal detachment and retinoschisis using handheld optical coherence tomography in stage 4 retinopathy of prematurity. JAMA Ophthalmol. (2020) 138(1):81–5. 10.1001/jamaophthalmol.2019.4796 31774474 PMC6902125

[64] Chiang MF Quinn GE Fielder AR Ostmo SR Paul Chan RV Berrocal A International classification of retinopathy of prematurity, third edition. Ophthalmology. (2021) 128(10):e51–68. 10.1016/j.ophtha.2021.05.031 34247850 PMC10979521

[65] Borrelli E Sarraf D Freund KB Sadda SR. OCT angiography and evaluation of the choroid and choroidal vascular disorders. Prog Retinal Eye Res. (2018) 67:30–55. 10.1016/j.preteyeres.2018.07.002 30059755

[66] Michalak SM Mangalesh S Shen LL McGeehan B Winter KP Sarin N Systemic factors associated with a thinner choroid in preterm infants. Ophthalmol Sci. (2021) 1(2):100032. 10.1016/j.xops.2021.100032 36249299 PMC9559969

[67] He Y Pettenkofer M Nittala MG Sadda SR Tsui I Chu A. Early postnatal oxygen exposure predicts choroidal thinning in neonates. Invest Ophthalmol Visual Sci. (2021) 62(9):23. 10.1167/iovs.62.9.23 PMC8297422 34269816

[68] Sola A Golombek SG Montes Bueno MT Lemus-Varela L Zuluaga C Domínguez F Safe oxygen saturation targeting and monitoring in preterm infants: can we avoid hypoxia and hyperoxia? Acta Paediatr. (2014) 103(10):1009–18. 10.1111/apa.12692 24838096 PMC4225465

[69] Askie LM. Meta-analysis of oxygenation saturation targeting trials: do infant subgroups matter? Clin Perinatol. (2019) 46(3):579–91. 10.1016/j.clp.2019.05.003 31345548

[70] Binenbaum G. Algorithms for the prediction of retinopathy of prematurity based on postnatal weight gain. Clin Perinatol. (2013) 40(2):261–70. 10.1016/j.clp.2013.02.004 23719309 PMC3692738

[71] Hellström A Ley D Hansen-Pupp I Niklasson A Smith L Löfqvist C New insights into the development of retinopathy of prematurity–importance of early weight gain. Acta Paediatr. (2010) 99(4):502–8. 10.1111/j.1651-2227.2009.01568.x 19878131

[72] Fieß A Christian L Kölb-Keerl R Knuf M Kirchhof B Muether PS Peripapillary choroidal thickness in former preterm and full-term infants aged from 4 to 10 years. Invest Ophthalmol Visual Sci. (2016) 57(15):6548–53. 10.1167/iovs.16-20128 27918828

[73] Li XQ Munkholm A Larsen M Munch IC. Choroidal thickness in relation to birth parameters in 11- to 12-year-old children: the Copenhagen child cohort 2000 eye study. Invest Ophthalmol Visual Sci. (2014) 56(1):617–24. 10.1167/iovs.14-15016 25358736

[74] Cai CX Go M Kelly MP Holgado S Toth CA. Ocular manifestations of poretti-boltshauser syndrome: findings from multimodal imaging and electrophysiology. Retin Cases Brief Rep. (2022) 16(3):270–4. 10.1097/ICB.0000000000000991 32195884 PMC7494654

[75] Grewal DS Tran-Viet D Vajzovic L Mruthyunjaya P Toth CA. Association of pediatric choroidal neovascular membranes at the temporal edge of optic nerve and retinochoroidal coloboma. Am J Ophthalmol. (2017) 174:104–12. 10.1016/j.ajo.2016.10.010 27793604

[76] Campos AG Lira RP Arantes TE. Optical coherence tomography of macular atrophy associated with microcephaly and presumed intrauterine Zika virus infection. Arq Bras Oftalmol. (2016) 79(6):400–1. 10.5935/0004-2749.20160112 28076569

[77] Ventura CV Ventura LO Bravo-Filho V Martins TT Berrocal AM Gois AL Optical coherence tomography of retinal lesions in infants with congenital Zika syndrome. JAMA Ophthalmol. (2016) 134(12):1420–7. 10.1001/jamaophthalmol.2016.4283 27832267

[78] Aygün FB Kadayifcilar S Lotfi Sadigh S Ozaltin F Eldem B. Multimodal imaging of severe oxalate retinopathy in a 20-month-old boy. Ophthalmic Surg Lasers Imaging Retina. (2022) 53(12):697–700. 10.3928/23258160-20221114-01 36547966

[79] Nguyen TP Ni S Liang G Khan S Wei X Skalet A Widefield optical coherence tomography in pediatric retina: a case series of intraoperative applications using a prototype handheld device. Front Med (Lausanne). (2022) 9:860371. 10.3389/fmed.2022.860371 35860728 PMC9289179

[80] Martin JH. Neuroanatomy text and atlas. New York: McGraw-Hill (2012).

[81] Tong AY El-Dairi M Maldonado RS Rothman AL Yuan EL Stinnett SS Evaluation of optic nerve development in preterm and term infants using handheld spectral-domain optical coherence tomography. Ophthalmology. (2014) 121(9):1818–26. 10.1016/j.ophtha.2014.03.020 24811961 PMC4145027

[82] Rothman AL Mangalesh S Chen X Toth CA. Optical coherence tomography of the preterm eye: from retinopathy of prematurity to brain development. Eye Brain. (2016) 8:123–33. 10.2147/eb.S97660 28539807 PMC5398750

[83] Pierrat V Marchand-Martin L Arnaud C Kaminski M Resche-Rigon M Lebeaux C Neurodevelopmental outcome at 2 years for preterm children born at 22–34 weeks’ gestation in France in 2011: EPIPAGE-2 cohort study. BMJ. (2017) 358:j3448. 10.1136/bmj.j3448 28814566 PMC5558213

[84] Rudanko SL Fellman V Laatikainen L. Visual impairment in children born prematurely from 1972 through 1989. Ophthalmology. (2003) 110(8):1639–45. 10.1016/S0161-6420(03)00498-6 12917186

[85] Garcia-Filion P Fink C Geffner ME Borchert M. Optic nerve hypoplasia in North America: a re-appraisal of perinatal risk factors. Acta Ophthalmol. (2010) 88(5):527–34. 10.1111/j.1755-3768.2008.01450.x 19141149 PMC3319088

[86] Lenzi Q Correia-Santos AM Lenzi-Almeida KC Boaventura GT. Flaxseed used since pregnancy by the mother and after weaning by the offspring benefits the retina and optic nerve development in rats. J Matern Fetal Neonatal Med. (2018) 31(5):625–32. 10.1080/14767058.2017.1293028 28282776

[87] Shen LL Mangalesh S Michalak SM Associations between systemic health and retinal nerve fibre layer thickness in preterm infants at 36 weeks postmenstrual age. Br J Ophthalmol. (2023) 107(2):242–7. 10.1136/bjophthalmol-2021-319254 34389548 PMC8858642

[88] Ng PC Ang IL Chiu RW Li K Lam HS Wong RP Host-response biomarkers for diagnosis of late-onset septicemia and necrotizing enterocolitis in preterm infants. J Clin Invest. (2010) 120(8):2989–3000. 10.1172/JCI40196 20592468 PMC2912182

[89] Ogura S Kurata K Hattori Y Takase H Ishiguro-Oonuma T Hwang Y Sustained inflammation after pericyte depletion induces irreversible blood-retina barrier breakdown. JCI Insight. (2017) 2(3):e90905. 10.1172/jci.insight.90905 28194443 PMC5291729

[90] Dinkin M. Trans-synaptic retrograde degeneration in the human visual system: slow, silent, and real. Curr Neurol Neurosci Rep. (2017) 17(2):16. 10.1007/s11910-017-0725-2 28229400

[91] Shah DK Doyle LW Anderson PJ Bear M Daley AJ Hunt RW Adverse neurodevelopment in preterm infants with postnatal sepsis or necrotizing enterocolitis is mediated by white matter abnormalities on magnetic resonance imaging at term. J Pediatr. (2008) 153(2):170–5; 175.e171. 10.1016/j.jpeds.2008.02.033 18534228

[92] Swanson JW Xu W Ying GS Pan W Lang SS Heuer GG Intracranial pressure patterns in children with craniosynostosis utilizing optical coherence tomography. Childs Nerv Syst. (2020) 36(3):535–44. 10.1007/s00381-019-04448-x 31848721

[93] Rufai SR Hisaund M Jeelani NUO McLean RJ. Detection of intracranial hypertension in children using optical coherence tomography: a systematic review. BMJ Open. (2021) 11(8):e046935. 10.1136/bmjopen-2020-046935 34380720 PMC8359522

[94] Avery RA Hwang EI Ishikawa H Acosta MT Hutcheson KA Santos D Handheld optical coherence tomography during sedation in young children with optic pathway gliomas. JAMA Ophthalmol. (2014) 132(3):265–71. 10.1001/jamaophthalmol.2013.7649 24435762 PMC4445404

[95] Banc A Stan C Florian IS. Optical coherence tomography as a marker of vision in children with optic pathway gliomas. Childs Nerv Syst. (2018) 34(1):51–60. 10.1007/s00381-017-3578-8 28844094

[96] Nuijts MA Degeling MH Stegeman I Schouten-van Meeteren AYN Imhof SM. Visual impairment in children with a brain tumor: a prospective nationwide multicenter study using standard visual testing and optical coherence tomography (CCISS study). BMC Ophthalmol. (2019) 19(1):220. 10.1186/s12886-019-1225-8 31706271 PMC6842490

[97] Go MS Barman NR Kelly MP House RJ Rotruck JC El-Dairi MA Overhead mounted optical coherence tomography in childhood glaucoma evaluation. J Glaucoma. (2020) 29(9):742–9. 10.1097/IJG.0000000000001567 32496465

[98] Ji X Wright T VandenHoven C MacKeen L McFarlane M Liu H Reliability of handheld optical coherence tomography in children younger than three years of age undergoing vigabatrin treatment for childhood epilepsy. Transl Vis Sci Technol. (2020) 9(3):9. 10.1167/tvst.9.3.9 32704429 PMC7347507

